# Implementing Dementia Friendly Initiatives in the Community: Perceptions of Stakeholders in the USA and China

**DOI:** 10.1093/geroni/igab046.1876

**Published:** 2021-12-17

**Authors:** Fei Sun, Ha Neul Kim, Lucas Prieto, Christian Conyers, Fredrika Opur

**Affiliations:** 1 Michigan State University, East Lansing, Michigan, United States; 2 University of Michigan, University of Michigan, Michigan, United States; 3 MSU, East Lansing, Michigan, United States

## Abstract

To address the exclusion of persons living with dementia (PWD), Dementia Friendly Initiatives (DFI) are being launched to build a friendly and supportive environment for PWD in the U.S.A, mainland China, and Taiwan. This study aims to identify the impact of DFI, the challenges DFI encountered, and strategies used to address such challenges within the COVID-19 context in American and Chinese societies. Individual interviews via Zoom with 9 stakeholders from the U.S.A, 8 from mainland China and one from Taiwan were transcribed for analyses. DFI have shown effectiveness in raising the public’s dementia awareness and engaging PWD in the community. COVID-19 pandemic posed the challenges of serving isolated PWD due to resources and attention shifted to COVID-19 prevention. Person-centered and technology-based means were used to deliver services for PWD during the pandemic. DFI in American and Chinese societies experienced similar sustainability challenges but showed resilience during the COVID-19 pandemic.

